# The role of three heat shock protein genes in the immune response to *Aeromonas hydrophila* challenge in marbled eel, *Anguilla marmorata*

**DOI:** 10.1098/rsos.160375

**Published:** 2016-10-19

**Authors:** Fenfei Liang, Guosong Zhang, Shaowu Yin, Li Wang

**Affiliations:** 1 Jiangsu Key Laboratory for Biodiversity and Biotechnology, College of Life Sciences, Nanjing Normal University, Nanjing, Jiangsu 210023, People's Republic of China; 2 Co-Innovation Center for Marine Bio-Industry Technology of Jiangsu Province, Lianyungang, Jiangsu 222005, People's Republic of China

**Keywords:** *Anguilla marmorata*, heat shock protein, mRNA expression, *Aeromonas hydrophila*, immune response

## Abstract

Heat shock proteins (HSPs) are highly conserved molecular chaperones that play critical roles in both innate and adaptive immunity. However, little information about HSPs from marbled eel *Anguilla marmorata* is known. In this study, the full-length *Amhsp90* (2527 bp), *Amhsp70* (2443 bp) and *Amhsc70* (2247 bp) were first cloned from *A. marmorata*, using rapid amplification of cDNA ends, containing open reading frames of 2181, 1932 and 1950 bp in length, and encoding proteins with 726, 643 and 649 amino acids, respectively. The deduced amino acid sequences of three *Amhsps* shared a high homology similarity with other migratory fish. Real-time fluorescent quantitative polymerase chain reaction was used to evaluate tissue-specific distribution and mRNA expression levels of three *Amhsps* subjected to infection with *Aeromonas hydrophila*. The mRNA expression of three *Amhsps* in eight tested tissues, namely liver, heart, muscle, gill, spleen, kidney, brain and intestine, of juvenile *A. marmorata* was evaluated to reveal the major expression distribution in liver, intestine, muscle and heart. After pathogen challenge treatments, mRNA transcriptions of three *Amhsps* revealed a significant regulation at various time points in the same tissue. All these findings suggest that *Amhsps* may be involved in the immune response in *A. marmorata*.

## 1. Introduction

*Anguilla marmorata* (*A. marmorata*) is a kind of typical catadromous migration fish [1], which is widely distributed in tropical and subtropical western Pacific areas, and cultivated in some European and Southeast Asian countries [2]. It is recognized as a high nutritional value and commercially important freshwater eel in Southeast Asia, and has been cultured in freshwater farms in China for many years [3,4]. During the period of farming, pathogenic bacterial infection often can cause a series of diseases during artificial cultivation, and lead to enormous economic loss [1].

Bacterial disease is the most common and harmful disease during the process of eel cultivation. *Aeromonas hydrophila* (*A. hydrophila*) is a major pathogenic bacterium present during freshwater farming of eel, which can cause many infectious diseases, such as haemorrhagic septicaemia, gill-rot disease and bacterial enteritis in European eel (*Anguilla anguilla*), Japanese eel (*Anguilla japonica*) and American eel (*Anguilla rostrata*) [5]. It can rapidly invade different tissues of eels to disrupt the expression of immune-relevant enzymes [6,7].

Heat shock proteins (HSPs) are extremely conserved proteins, and exist widely from bacteria to plants, mammals, prokaryotes, yeast and fish [8]. As molecular chaperones, HSPs play a key role in response to potential stress conditions, including oxidative stress, heat stress, heavy metal contamination and bacterial infection [9]. According to different molecular masses, HSPs are classified into several families, including *hsp100, hsp90, hsp70, hsp60* and other HSPs with low molecular masses [10]. *Hsp70* and *hsp90* family was widely studied in eukaryotes. *Hsp90* family has two major cytosolic subtypes such as *hsp90-alpha* and *hsp90-beta*. Well-recognized members of the *hsp70* multigene family are two closed cytosolic forms: cognate *hsc70* and inducible *hsp70* [11].

The full lengths of *hsp* genes and corresponding immune responses have been identified in several fish species, which includes the responses of silver sea bream (*Sparus sarba*) to *Vibrio alginolyticus*, miiuy croaker (*Miichthys miiuy*) to *Vibrio anguillarum*, grass carp (*Ctenopharyngodon idella*) to lipopolysaccharide and humphead snapper (*Lutjanus sanguineus*) to *Vibrio harveyi* infection [12–15]. However, the mRNA expression pattern of *hsp* genes after being challenged with *A. hydrophila* has been rarely reported. The comparative classical studies were concentrated on Wuchang bream (*Megalobrama amblycephala*) and walking catfish (*Clarias macrocephalus*), mandarin fish (*Siniperca chuatsi*) and *Botia reevesae* [5,16–18]. Although different expression profiles among *hsp* genes have been observed in many species, whether the difference is observed in other fish species under the same challenge and what the expression profiles are during the challenge with *A. hydrophila* are still unclear.

In this study, we first, to the best of our knowledge, reported the full-length cDNAs of *Amhsp90*, *Amhsp70* and *Amhsc70* cloned from *A. marmorata*. The deduced amino acid sequences were compared with other known *hsps* from other fish species. The expression levels of three *Amhsps* in various tissues were investigated, and the expression patterns in liver, muscle, intestine and heart challenged with *A. hydrophila* were explored. All of these studies contribute to a better understanding of the innate immunity of *A. marmorata* and provide a useful theoretical rationale to clarify the underlying mechanism of *hsps* in eels.

## 2. Material and methods

### 2.1. Fish and infection

Juvenile *A. marmorata* with body weight of 15.5 ± 3.3 g were obtained from Wenchang Jinshan Eel Technology Limited Company in Wenchang, Hainan, China (approval number: National Fishery Resources and Environmental Protection 2004; 13). All samples used in this study were approved by the Animal Ethics Committee of Nanjing Normal University (permit no. SYXK2015-0028). The fish were cultured in 120 l aerated plastic tanks at 24°C for three weeks with one time a day feeding of artificially formulated feed before testing. *A. hydrophila* (ATCC7966, Microbial Culture Collection Center, Beijing, China) were inoculated in broth bouillon and incubated in a shaker at 28°C for 24 h. The bacteria were collected and diluted with 0.85% NaCl to a final concentration of 1.0 × 10^8^ CFU ml^−1^.

*Anguilla marmorata* were divided into three groups: the blank group for tissue distribution, the control group and the experiment group. All treatments were conducted in triplicate; in addition, each experiment was operated with three fish mixed samples (*n* = 9). Tissue samples, including liver, heart, muscle, gill, spleen, kidney, brain and intestine, were collected from three non-infected fish as the blank group. The fish in the experiment group were intramuscularly injected with 0.1 ml of *A. hydrophila* (1.0 × 10^8^ CFU ml^−1^ for each individual), whereas the control fish were injected with 0.1 ml of 0.85% NaCl. After treatment, the fish were returned to the culture tanks, and the tissue samples (liver, muscle, intestine and heart) from every three fish per group were randomly collected at 1, 3, 6, 12, 24, 48 and 72 h post-injection.

### 2.2. RNA extraction and cDNA synthesis

Total RNA was isolated from the samples of all untreated and treated fish at each sampling time point to explore tissue-specific distribution and the effect of pathogen challenge on mRNA expression levels of *Amhsp90*, *Amhsp70* and *Amhsc70*. Liver, kidney, spleen, gill, muscle, heart, brain and intestine samples were collected from *A. marmorata* for RNA extraction. Total RNA was extracted using rapid extraction kit (BioTeke, Beijing, China). The quality of RNA integrity and cDNA production by reverse transcription was checked with 1.0% agarose gel electrophoresis. Reverse transcription templates were synthesized using HiScript™ QRT SuperMix (Vazyme, NJ) according to the manufacturer's protocols. To perform the rapid amplification of cDNA ends (RACE), we used the universal primer A mix primer and gene-specific primers, and the gene cloning was conducted using Clontech Advantage 2 PCR kit from Takara (Dalian, China). The amplified products were cloned into pMD18-T vectors and sequenced by Beijing Genomics Institute (Beijing, China). The detailed procedures were performed according to the manufacturer's instructions. All primers are listed in table 1.

**Table 1.** Primers used for gene cloning and expression analysis (F, forward primer; R , reverse primer; GSP, gene-specific primer).

application	primer names	sequences (5'–3′)
5′-RACE	Amhsp90-5′-GSP1	TCCGATGCCTGTGTC
	Amhsp90-5′-GSP2	CATCTGAGGAGTTGGAGATGA
	Amhsp90-5′-GSP3	GATCTCTTTGTTGGAGTAGAAAG
3′-RACE	Amhsp90-3′-GSP1	CTTGAGATTAATCCYGAGCACCCCAT
	Amhsp90-3′-GSP2	CTACMGGATGATYAARCTTGGCCTGGG
ORF	Amhsp90-QC-F	CAAGATAACTACATGTGACCAGC
	Amhsp90-QC-R	TCAGTCGACTTCCTCCATCCTGG
qRT-PCR	Amhsp90-RT-F	AGAGCGTGATAAGGAGGTGAG
	Amhsp90-RT-R	TGTCATCTGGGTTTCTTGTCCA
5′-RACE	Amhsp70-5′-GSP1	CTCGGTGTCTGTGAAG
	Amhsp70-5′-GSP2	GTTCTGTTGCCCTGGTCGTT
	Amhsp70-5′-GSP3	CACACCCACACAGGAGTAGG
3′-RACE	Amhsp70-3′-GSP1	GGTTATCGCCTGGCTGGAGGACAAT
	Amhsp70-3′-GSP2	GTGTGTAATCCCATCATCGCCAAGC
ORF	Amhsp70-QC-F	AAGCCTGGCGGAAGGTCGAG
	Amhsp70-QC-R	TTAATCCACCTCCTCAATGGTG
qRT-PCR	Amhsp70-RT-F	AATGATGGCGGTCGTCCAAA
	Amhsp70-RT-R	TGAAATTGTACCGGCGTCCT
5′-RACE	Amhsc70-5′-GSP1	CAATCAGCCGCTCAGT
	Amhsc70-5′-GSP2	TGTGGTCCTGTTTCCCTGAT
	Amhsc70-5′-GSP3	CATGCTGGAAGACACCTACG
3′-RACE	Amhsc70-3′-GSP1	CCTACGCATTCAACATGAAATCCACCG
	Amhsc70-3′-GSP2	GGAAGAGTTTGAGCATCAACAGCAGGA
ORF	Amhsc70-QC-F	GAAACAGCTCCATTGTACGGTG
	Amhsc70-QC-R	TTAATCGACTTCCTCAATAGTTGGC
qRT-PCR	Amhsc70-RT-F	AGCTGTCGCTTATGGTGCAG
	Amhsc70-RT-R	AGGTCTGGGTCTGTTTTGTGG
	β-actin-RT-F	GCAGATGTGGATCAGCAAGC
	β-actin-RT-R	ACATTGCCGTCACCTTCATG

### 2.3. Sequence analysis and phylogenetic analysis

The sequences were obtained from polymerase chain reaction amplification, the ORF and RACE were assembled using DNA star software to assemble the full-length cDNA, and the full-length cDNA sequence was subjected to homology analysis. Similarity searching of amino acid sequences was conducted with BLAST in NCBI (http://www.ncbi.nlm.nih.gov/BLAST/). The isoelectric points of deduced proteins were predicted using ExPASy (http://www.au.expasy.org/). Translation of cDNAs and multiple sequence alignments was conducted with DNAMAN software (Lynnon Biosoft, Quebec, Canada), and characteristic motifs and domains were predicted using the simple modular architecture research tool (SMART; http://smart.embl-heidelberg.de/) and InterProScan (http://www.ebi.ac.uk/interpro/). To examine the evolutionary relationships among the *hsp90* and *hsp70* family members in other species, a phylogenetic tree of different vertebrate *hsp* genes based on amino acid sequences was constructed by the neighbour-joining method and bootstrapped for 1000 replicates using MEGA v. 5 program (http://www.megasoftware.net/megamac.php).

### 2.4. Tissue distribution and mRNA expression of heat shock proteins

The real-time fluorescent quantitative polymerase chain reaction (RT-qPCR) method with β-actin as an internal control was used to explore the mRNA expression levels of *Amhsp90*, *Amhsp70* and *Amhsc70* in various tissues, including liver, heart, muscle, gill, spleen, kidney, brain and intestine, of untreated *A. marmorata*. RT-qPCR was performed following the manufacturer's protocol of the kit of SYBR Green Master (Roche, Basel, Switzerland). The primers for RT-qPCR (e.g. *Amhsp90*-RT-F/*Amhsp90*-RT-R) are listed in table 1. The experiments were carried out in triplicate with a total volume of 20 µl in ABI stepone™ plus (Applied Biosystems, USA), containing 10 µl of SYBR Green Master, 4 µl of cDNA (dilution to 5 ng µl^−1^) and 3 µl of each forward or reverse primer (2 µmol l^−1^). RT-qPCR was programmed at 95°C for 10 min, followed by 40 cycles of 95°C for 15 s, and 55°C for 1 min, and a final extension at 72°C for 60 s. To confirm the specificity of the amplification, the dissociation curve was analysed for amplified products to ensure an obvious amplification peak. The expression level of *Amhsps* was calculated by 2^−△△C^_T_ method and subjected to statistical analysis [19]. Similarly, RT-qPCR was also used to explore mRNA expression of *Amhsps* in liver, muscle, intestine and heart after being challenged with *A. hydrophila*.

### 2.5. Statistical analysis

Statistical analysis was performed using SPSS 19.0. The relevant values in this study were analysed through one-way analysis of variance followed by Tukey's test. Statistical significance was considered at *p* < 0.05, and highly significant difference was considered at *p* < 0.01. All data were expressed as mean standard errors (s.e.) in terms of relative mRNA expression.

## 3. Results

### 3.1. Identification and characterization of *Amhsp90*, *Amhsp70* and *Amhsc70* cDNA sequences

**Figure 1.**
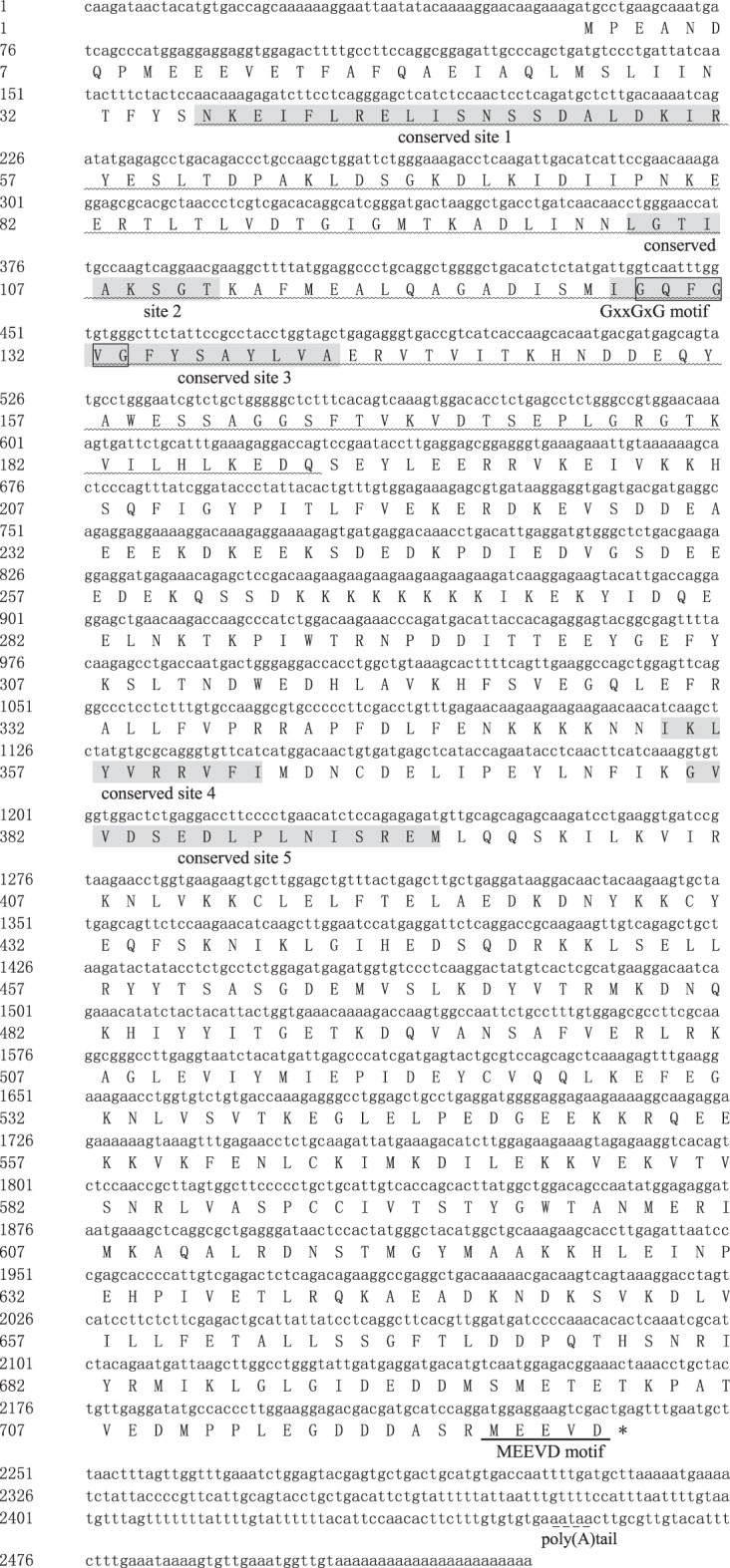
Nucleotide and deduced amino acid sequences of *Amhsp90* (GenBank accession no. KT274762). The ATPase domain of *Amhsp90* is highlighted as a wavy line. The protein family signature is shown in shaded regions. The ‘GxxGxG’ motif is labelled in the box. The ‘MEEVD’ motif is indicated by an underline. The stop codon is labelled as an asterisk. The polyadenylation signal (aataa) is shown in short dashed line.

The *Amhsp90* was deposited in the GenBank database with an accession number of KT274762 and named as *Amhsp90*. The nucleotide and deduced amino acid sequences of the full-length cDNAs are shown in figure 1. This cDNA with an ORF of 2181 bp encoding 726 amino acid residues displays a calculated molecular mass of 83.6 kDa and a theoretical isoelectric point (PI) of 4.97. The 5′ and 3′ untranslated regions (UTRs) were 58 and 288 bp, respectively, with a canonical polyadenylation signal sequence of AATAAA and a poly (A) tail (figure 1). A typical histidine kinase-like ATPase domain was located at 36–190 aa of *Amhsp90* using SMART analysis (figure 1). Five conserved *Amhsp90* protein family signatures were detected in the deduced amino acid (aa) sequences (figure 1): NKEIFLRELISNSSDALDKIR (36–56 aa), LGTIAKSGT (103–111 aa), IGQFGVGFYSAYLVA (127–141 aa), IKLYVRRVFI (354–363 aa) and GVVDSEDLPLNISREM (380–395 aa). In the significant motif 3, a conserved ‘GxxGxG’ motif (128–133 aa) was observed, and wrapped around ATP in the tertiary structure [20]. Signal peptide and transmembrane domain were not detected in *Amhsp90*. The conserved ‘MEEVD’ motif was located at the C terminus of *Amhsp90*.

**Figure 2.**
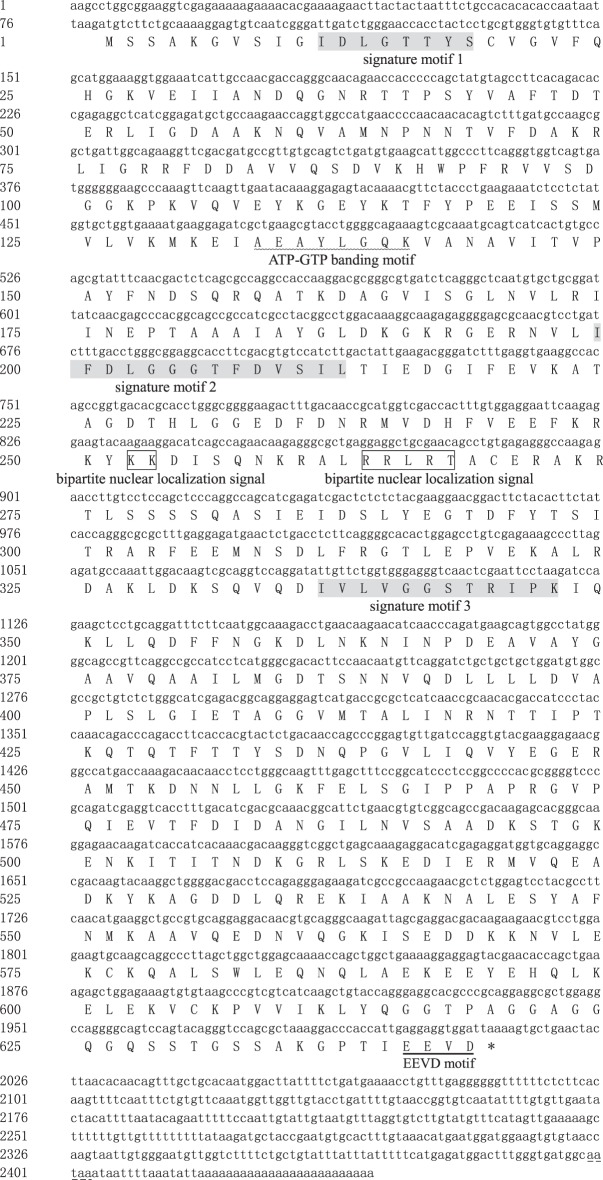
Nucleotide and deduced amino acid sequences of *Amhsp70* (GenBank accession no. KT274761). The ATPase domain of *Amhsp70* is highlighted as a wavy line. The protein family signature is shown in shaded regions. The putative bipartite nuclear localization signals (KK and RRLRT) are shown in a box. The ‘EEVD’ motif is indicated by an underline. The stop codon is labelled as an asterisk. The polyadenylation signal (aataa) is shown in a short dashed line.

The cytoplasmic *hsp70* family consists of constitutive form *hsc70* and inducible form *hsp70* subfamilies. The full-length cDNA of *Amhsp70* (accession no. KT274761) consisted of 79 bp 5′-UTR, 432 bp 3′-UTR with a poly (A) tail, and 1932 bp ORF encoding 643 amino acids (figure 2). The molecular mass of the deduced protein was approximately 70.50 kDa with an estimated PI of 5.44. The predicted amino acid sequence of *Amhsp70* contained an ATP/GTP-binding site motif A [16], a putative bipartite nuclear localization signal (KK and RRLRT), three conserved sites of the *hsp70* family (IDLGTTYS, IFDLGGGTFDVSIL and IVLVGGSTRIPKIQK) and the cytoplasmic characteristic motif EEVD (figure 2) [21,22].

**Figure 3.**
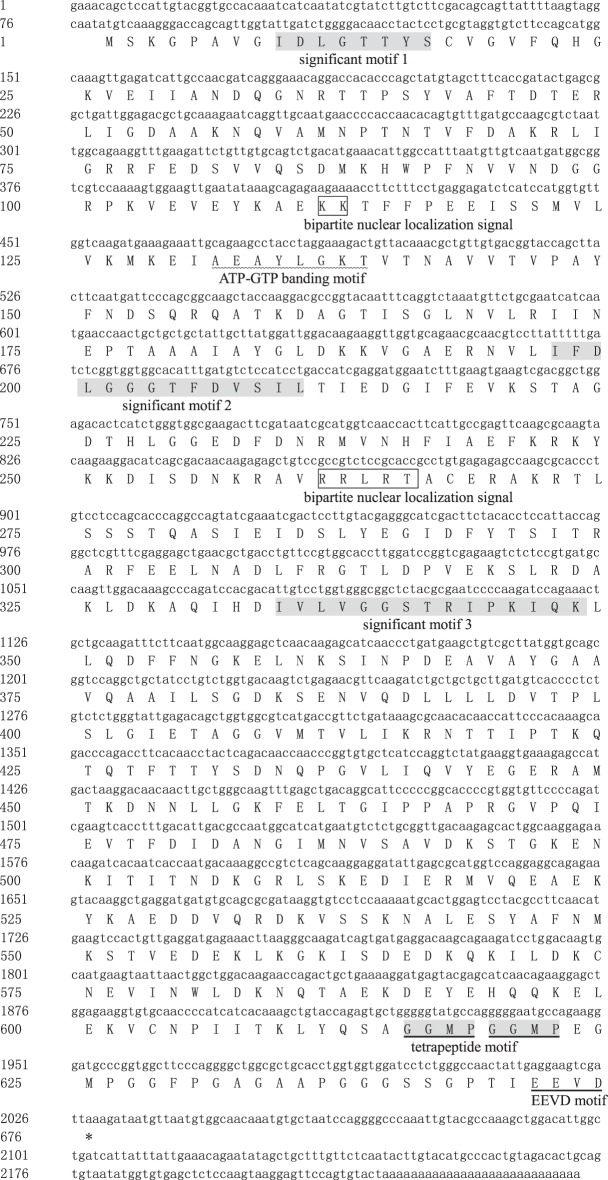
Nucleotide and deduced amino acid sequences of *Amhsc70* (GenBank accession no. KT274760). The ATPase domain of *Amhsc70* is highlighted as a wavy line. The protein family signature is shown in shaded regions. The putative bipartite nuclear localization signals (KK and RRLRT) are shown in a box. The ‘EEVD’ motif is indicated by an underline. Two consecutive repeats of tetrapeptide motif GGMP are shown in a shaded region and underline. The stop codon is labelled as an asterisk.

The cDNA of *Amhsc70* (accession no. KT274760) contained 79 bp of 5′ UTR, 1950 bp of ORF encoding 649 amino acids, and followed by 218 bp of 3′ UTR with a poly (A) tail (figure 3). The calculated molecular weight of the deduced peptide was 71.21 kDa, and the predicted theoretical PI was 5.28. At the carboxyl terminal region, *Amhsc70* contained three conserved sites such as *Amhsp70*, the cytoplasmic characteristic motif EEVD [21,22] and two consecutive repeats of the tetrapeptide motif GGMP (615–622 aa; figure 3) [16,23].

### 3.2. Multiple sequence alignment and phylogenetic analysis

**Figure 4.**
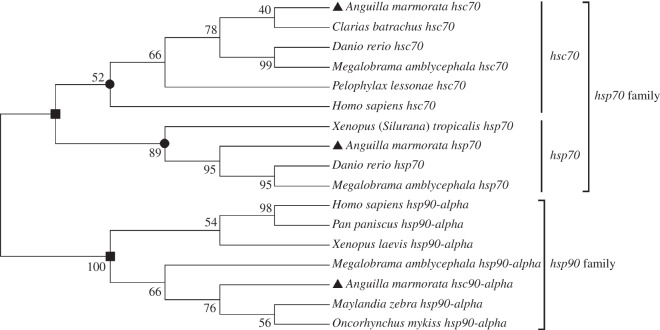
Phylogenetic tree showing the relationship of *Amhsp90*, *Amhsp70* and *Amhsc70* amino acid sequences relative to other *hsp90/hsp70* family members from other species. The tree was constructed with the neighbour-joining method by MEGA 5 software. The number at each node indicates the percentage of bootstrapping after 1000 replications. The sequences were taken from the GenBank sequence databases. *Clarias batrachus hsc70* (AGI03834.2), *Danio rerio hsc70* (AAB03704.1), *Megalobrama amblycephala hsc70* (ACS74754), *Pelophylax lessonae hsc70* (ACY69995.1), *Homo sapiens hsc70* (AAK17898.1), *Xenopus (Silurana) tropicalis hsp70* (AAI55368.1), *Danio rerio hsp70* (AAH56709.1), *Megalobrama amblycephala hsp70* (ACG63706.2), *Homo sapiens hsp90-alpha* (AAI21063.1), *Pan paniscus hsp90-alpha* (XP_008957357.1), *Xenopus laevis hsp90-alpha* (NP_001085598.1), *Megalobrama amblycephala hsp90-alpha* (AGV06257.1), *Maylandia zebra hsp90-alpha* (XP_004542147.1), *Oncorhynchus mykiss hsp90-alpha* (CDQ81679.1).

*Amhsp90* amino acid sequence showed high identity with *hsp90-alpha* in *Salmon salar* (90%), *Danio rerio* (86%); *Amhsp70* and *Amhsc70* showed high identity with *hsp70* and *hsc70* in *S. salar* (87%, 96%) and *D. rerio* (90%, 96%). The alignment analysis between *Amhsp70* and *Amhsc70* amino acid sequences showed the identity of 83.41%. Conserved sequence and characteristic motifs were identified in the deduced amino acid sequences of *Amhsp90*, *Amhsp70* and *Amhsc70*. The alignment results showed that the amino acid sequences of *hsp90* family and *hsp70* family have significant differences. To examine the relationships among *hsp90*, *hsp70* and *hsc70*, the phylogenetic tree was established by MEGA v. 5.0 based on the neighbour-joining method through amino acid sequences. Different *hsp90*, *hsp70* and *hsc70* family members were selected from other vertebrate species, respectively. The phylogenetic tree showed that these proteins were divided into two major groups. One group comprised *hsp70* family, and the other group contained *hsp90* family. All constitutive form *hsc70* and inducible form *hsp70* were clustered in the branch of *hsp70* family (figure 4).

### 3.3. Tissue-specific distribution of *Amhsp* genes

**Figure 5.**
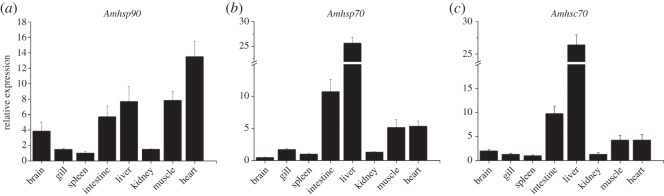
Tissue distribution of *Amhsp90* (*a*)*, Amhsp70* (*b*) and *Amhsc70* (*c*) genes from *A. marmorata* in brain, gill, spleen, intestine, liver, kidney, muscle and heart was explored using RT-qPCR methods. Each sample was run in triplicate. Deviation bars are the standard errors. The *A. marmorata* β-actin gene was used as an internal control to calibrate the cDNA template for all samples.

Tissue-specific distribution analysis using RT-qPCR method showed that three *hsp* mRNAs were ubiquitously expressed in all detected tissues of liver, heart, muscle, gill, spleen, kidney, brain and intestine. The mRNA transcripts of three *Amhsps* were expressed at a relatively high level in liver, intestine, muscle and heart, at the lowest level in spleen and at a moderate level in other examined tissues (figure 5).

### 3.4. Expression of *Amhsps* in response to *Aeromonas hydrophila* injection

**Figure 6.**
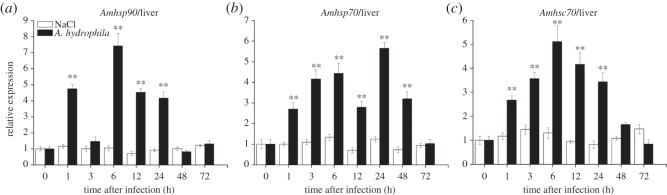
Relative mRNA expression of *Amhsp90* (*a*), *Amhsp70* (*b*), *Amhsc70* (*c*) after *A. hydrophila* challenge was measured by RT-qPCR. Liver collected from *A. marmorata* was injected with 0.85% NaCl or *A. hydrophila* at 0, 1, 3, 6, 12, 24, 48 and 72 h. The mRNA levels of *Amhsps* were analysed and standardized according to the β-actin mRNA levels. Deviation bars represent the standard errors of three experiments at each time point. Asterisks indicate significant differences (*p < 0.05, **p < 0.01) when compared with values from the control group.

**Figure 7.**
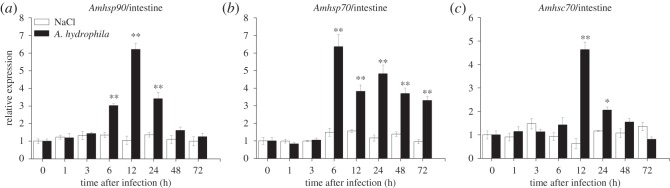
Relative mRNA expression of *Amhsp90* (*a*), *Amhsp70* (*b*), *Amhsc70* (*c*) after *A. hydrophila* challenge was measured by RT-qPCR. Intestine collected from *A. marmorata* was injected with 0.85% NaCl or *A. hydrophila* at 0, 1, 3, 6, 12, 24, 48 and 72 h. The mRNA levels of *Amhsps* were analysed and standardized according to the β-actin mRNA levels. Deviation bars represent the standard errors of three experiments at each time point. Asterisks indicate significant differences (*p < 0.05, **p < 0.01) when compared with values from the control group.

**Figure 8.**
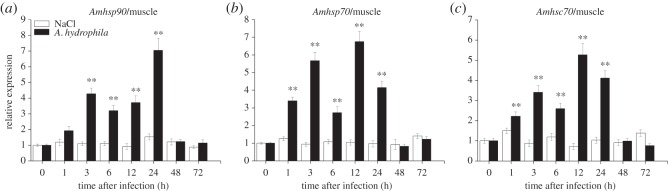
Relative mRNA expression of *Amhsp90* (*a*), *Amhsp70* (*b*), *Amhsc70* (*c*) after *A. hydrophila* challenge was measured by RT-qPCR. Muscle collected from *A. marmorata* was injected with 0.85% NaCl or *A. hydrophila* at 0, 1, 3, 6, 12, 24, 48 and 72 h. The mRNA levels of *Amhsps* were analysed and standardized according to the β-actin mRNA levels. Deviation bars represent the standard errors of three experiments at each time point. Asterisks indicate significant differences (*p < 0.05, **p < 0.01) when compared with values from the control group.

**Figure 9.**
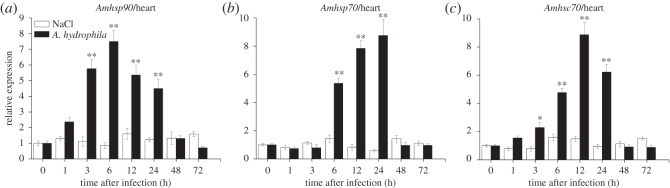
Relative mRNA expression of *Amhsp90* (*a*), *Amhsp70* (*b*), *Amhsc70* (*c*) after *A. hydrophila* challenge was measured by RT-qPCR. Heart collected from *A. marmorata* was injected with 0.85% NaCl or *A. hydrophila* at 0, 1, 3, 6, 12, 24, 48 and 72 h. The mRNA levels of *Amhsps* were analysed and standardized according to the β-actin mRNA levels. Deviation bars represent the standard errors of three experiments at each time point. Asterisks indicate significant differences (*p < 0.05, **p < 0.01) when compared with values from the control group.

The temporal expression profile of *Amhsp90* was observed after bacterial challenge. In liver, the expression level of *Amhsp90* revealed a rapid upregulation within 1 h after the challenge with *A. hydrophila* and a peak level at 6 h, and then exhibited a decreasing trend from 48 to 72 h (figure 6*a*). In intestine, *Amhsp90* reached the highest level at 12 h (figure 7*a*). In muscle and heart, the expression of *Amhsp90* was rapidly upregulated from 3 to 24 h and reached the highest level at 24 and 6 h, and then dropped rapidly (figures 8*a* and 9*a*).

Upregulated expression of *Amhsp70* mRNA from 1 h post-challenge in liver and muscle was observed (figures 6*b* and 8*b*). After treatment, the inducible *Amhsp70* showed a significantly high expression at 6 h in intestine (figure 7*b*) and heart (figure 9*b*). From 6 to 72 h, *Amhsp70* showed a highly significant difference in intestine, but it showed a highly significant difference in heart only between 6 and 24 h. In heart, the expression level of *Amhsp70* was rapidly upregulated from 6 to 24 h. At 24 h, the expression reached the peak level, and then levelled off at 48 h.

The expression of *Amhsc70* mRNA presented a fluctuating trend in muscle (figure 8*c*) and liver (figure 6*c*). In intestine, *Amhsc70* mRNA expression level basically remained unchanged when compared with that in the control group, and reached the maximum level at 12 h (figure 7*c*). In heart, *Amhsc70* showed a significant upregulation at a middle phase, and then rapidly decreased in a short time (figure 9*c*).

## 4. Discussion

HSPs are stress response proteins as a ‘dangerous signal’ to protect the immune system and the immune cells involved in the protection of cytoplasm components, including all kinds of biological factors, such as bacterial infection [24]. It can be used as the immune system to identify the important antigens for two reasons: first, the mRNA expression of *hsps* in most organisms revealed an obvious increase in the process of immune response when pathogens are engulfed by macrophages, in order to protect the organism and to maintain life force. Second, HSP is highly conserved, and the immune system can easily identify these highly conservative molecules. When bacteria invade an organism, the organism may release certain cell toxins, and promote intracellular cytokine synthesis and secretion caused by the variation of protein or polypeptide chain fragments. These abnormal proteins can be induced by *hsp* genes in cells with high expression efficiency [25].

**Figure 10.**
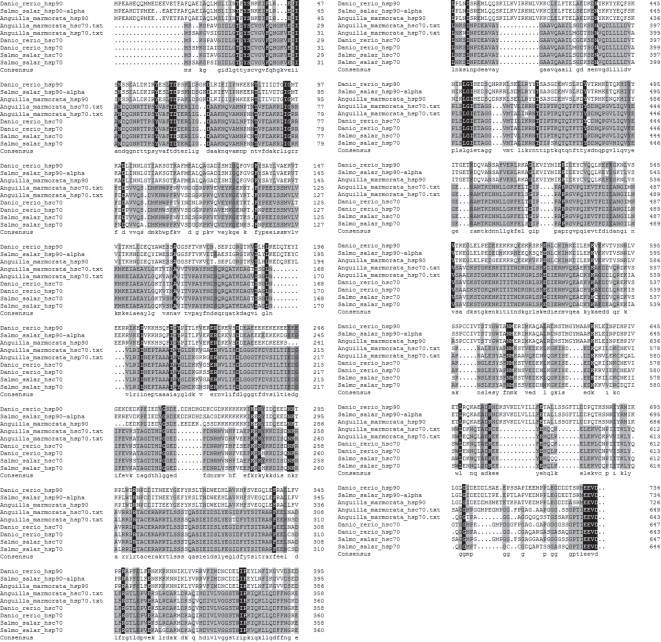
Multiple alignment of the deduced amino acid sequences: *Salmo sala hsp90-alpha* (NP_001167173.1), *Danio rerio hsp90-alpha* (NP_571403.1), *Salmo sala hsp70* (ACI34374.1), *Danio rerio hsp70* (AAH56709.1), *Salmo sala hsc70* (ACN11074.1), *Danio rerio hsc70* (AAH45841.1). Identical and similar amino acid residues are indicated with black and grey background, respectively. Gaps were introduced to maximize the alignment.

In this study, we obtained full-length cDNAs of *Amhsp90*, *Amhsp70* and *Amhsc70* of *A. marmorata* for the first time. They are similar to most of the known HSPs in teleost fish. Moreover, multiple sequence alignment results also indicated that *hsps* were highly conserved, suggesting that *Amhsps* may share a similar function with other known *hsps* (figure 10). In the phylogenetic tree, three *Amhsps* were clustered together with teleost. *Amhsp70* and *Amhsc70* were clustered into a major branch and all belonged to *hsp70* family (figure 4). The topological structure displayed in the phylogenetic tree is in good agreement with traditional taxonomy. The molecular information of *Amhsps* will be more useful for further exploring the expression of *hsp* genes, such as thermal stress, and this gene sequence information expands the gene database, and provides a theoretical basis for further studies on other *hsp* genes in other teleost fishes.

The *Amhsps* exhibited a similar gene expression pattern in different tissues. A broad tissue distribution of *hsp* genes was observed in *A. marmorata*, which revealed high expression in liver, intestine, heart and muscle. Specifically, *Amhsp70* and *Amhsc70* were expressed at high levels in liver, and *Amhsp90* mRNA expression was the highest level in heart. In comparison with *M. amblycephala, S. senegalensis, S. ocellatus* and *O. niloticus*, there were some different expression patterns with tissue-specific distribution [26–29]. Therefore, the distribution of *hsp* genes is varied in different tissues owing to different species. There is a tissue- or species-specific profile in response to the challenge with *A. hydrophila*.

According to the tissue-specific distribution of three *hsp* genes, four tissues (liver, muscle, intestine and heart) were selected as candidates. In liver, *Amhsp90*, *Amhsp70* and *Amhsc70* were rapidly upregulated within 1 h of *A. hydrophila* challenge and peaked at 6, 24 and 6 h (*p* < 0.01; figure 6). The experimental results showed that three *Amhsps* were sensitive to bacterial stimulation in liver, especially at the early stage following injection. The initial upregulation may be due to bacterial toxicity, and *Amhsps* gene could be activated quickly and transferred to the cytosol [30], which was in accordance with the expression patterns of *ScHSP70s* following *A. hydrophila* infection and *M. amblycephala* [12,31]. In the whole experiment process, the upregulation of *hsps* may be a protective mechanism, because *hsps* could bind to the damaged or misfolded proteins to restore their original structures [24,32].

In muscle, three *Amhsps* showed a similar dynamic trend. *Amhsp70* and *Amhsc70* mRNA levels reached a maximum at 12 h and then gradually decreased after bacterial challenge. However, *Amhsp90* significantly increased until the challenge for 24 h and then sharply reduced. In *C. macrocephalus*, *hsc70*-1 was nearly constant and *hsc70*-2 revealed a continuous increase. Once the intramuscular injection is applied, bacteria can immediately affect muscle lesions, and the high levels of these three *Amhsps* in muscle may reflect cell protection of *hsps* [5]. In liver and muscle tissues, three *Amhsp* genes showed different expression patterns, which may be due to liver tissue as the most important metabolic organ and defence organ with rapid mRNA expression of *Amhsps* with more intensity in the early phase during 72 h of infection. However, muscle tissue is located under the skin. When the fish were injected with *A. hydrophila*, it was easy to cause damage of skin and muscle. Therefore, the mRNA expression of *Amhsp* genes increased with an increase in swimming time, the contact surface of the wound may be enlarged, and the time point of high expression of *Amhsps* in muscle is relatively delayed when compared with liver.

There are very few reports about the expression patterns of intestinal mRNA owing to bacterial infection in fish. A surprising discovery of this study is that the mRNA expression levels of *Amhsp90* and *Amhsc70* reveal the peak level at 12 h (*p *< 0.01). The expression level of *Amhsp90* exhibits a rapid increase to the peak level at 12 h (*p *< 0.01) post-infection and a sequential decrease. In comparison with *Amhsp90*, *Amhsc70* mRNA expression reveals a sharp rise at 12 (*p *< 0.01) and 24 h (*p* < 0.05), and there is no significant change at other time points. At the same time, *Amhsp70* mRNA expression from 6 (*p *< 0.01) to 24 h (*p *< 0.01) shows an increasing trend. In contrast, in channel catfish, *hsp90* does not show a significant fold change in intestine at 3 h, 24 h and 3 days after *Edwardsiella ictaluri* infection [33]. This study suggested that three *Amhsps* played an important role in immune stress. Likewise, a clear time-dependent mRNA expression pattern of *Amhsp90* in heart was also observed when infected with *A. hydrophila*. *Amhsp70* and *Amhsc70* mRNA expression exhibited an increase at the middle phase, reached the peak at 24 (*p *< 0.01) and 12 h (*p* < 0.01), and began to decrease. According to an early report, *hsp60* has a significant increase at 4 h after *A. hydrophila* challenge in heart of grass carp.

In teleost fish, reports regarding heart and intestinal immune mechanisms associated with *hsps* are rather few. Our results suggest that *Amhsps* plays an important role in the intestine and heart of flower eel. Intestine digestive tube contains diffused lymphoid tissue, lymphocytes, macrophages and plasma cells, which are involved in immune defence. When intestinal mucosa was infected by bacteria after the stimulation of lymphoid tissue within the mucosal immune response, then endocrine immune globulin was produced in the digestive tube to prevent bacterial invasion in the digestive tract, thus regulating the compositions of the intestinal mucosal immune system [34]. Therefore, the infection of bacteria caused interesting expression patterns. At the same time, HSP is an important stress protein to protect myocardial cells against myocardial injury, can participate in the repair and restoration of ion channel redox balance, reduce the release of oxygen free radicals, and can also be used as an antioxidant for free radicals as a molecular chaperone to protect cells from damage [35]. Thus, in heart, the expression of *Amhsps* is very concentrated and intense.

In general, this is the first time of cloning cDNAs of *Amhsp90*, *Amhsp70* and *Amhsc70*. Bioinformatic analysis has confirmed that three *Amhsps* belong to *hsp90* family and *hsp70* family, respectively, and are ubiquitously expressed in eight tested tissues. We have also demonstrated that three *Amhsps* present more rapid and sensitive expression in liver and muscle after *A. hydrophila* challenge, whereas a relatively delayed sensitivity was observed in intestine and heart. These *Amhsp* genes may be involved in the regulation of *A. hydrophila* response in *A. marmorata*.

## Supplementary Material

1. Tissue distribution of original data

## Supplementary Material

2. The original gene expression data including four tissue

## Data accessibility

Data file on tissue distribution and gene expression from this study can be found in the electronic supplementary material.

## DNA deposition

The following information regarding the deposition of DNA sequences is supplied: GenBank accession nos. KT274760-KT274762.
